# Climate Change and Health on the U.S. Gulf Coast: Public Health Adaptation is Needed to Address Future Risks

**DOI:** 10.3390/ijerph120809342

**Published:** 2015-08-11

**Authors:** Elisaveta P. Petkova, Kristie L. Ebi, Derrin Culp, Irwin Redlener

**Affiliations:** 1 National Center for Disaster Preparedness, Earth Institute, Columbia University, Suite 303, 215 West 125th Street, New York, NY 10027, USA; E-Mails: dculp@verizon.net (D.C.); ir2110@columbia.edu (I.R.); 2 Department of Global Health, University of Washington, Seattle, WA 98105, USA; E-Mail: krisebi@uw.edu; 3 Department of Health Policy and Management, Mailman School of Public Health, Columbia University, New York, NY 10032, USA

**Keywords:** climate change, health, Gulf Coast, adaptation, preparedness

## Abstract

The impacts of climate change on human health have been documented globally and in the United States. Numerous studies project greater morbidity and mortality as a result of extreme weather events and other climate-sensitive hazards. Public health impacts on the U.S. Gulf Coast may be severe as the region is expected to experience increases in extreme temperatures, sea level rise, and possibly fewer but more intense hurricanes. Through myriad pathways, climate change is likely to make the Gulf Coast less hospitable and more dangerous for its residents, and may prompt substantial migration from and into the region. Public health impacts may be further exacerbated by the concentration of people and infrastructure, as well as the region’s coastal geography. Vulnerable populations, including the very young, elderly, and socioeconomically disadvantaged may face particularly high threats to their health and well-being. This paper provides an overview of potential public health impacts of climate variability and change on the Gulf Coast, with a focus on the region’s unique vulnerabilities, and outlines recommendations for improving the region’s ability to minimize the impacts of climate-sensitive hazards. Public health adaptation aimed at improving individual, public health system, and infrastructure resilience is urgently needed to meet the challenges climate change may pose to the Gulf Coast in the coming decades.

## 1. Introduction

The impacts of climate change on human health have been documented globally and in the United States [1]. Numerous studies project greater morbidity and mortality from direct exposure to extreme weather events, as well as greater health risks due to decreased air quality, water-borne diseases, and other infectious diseases [1,2,3,4]. Climate variability and change may also present threats to mental health and social stability, potentially leading to increased conflict, violence, and widespread migration away from areas that can no longer provide sufficient food, water, and shelter for the current populations [5]. Coastal areas are particularly vulnerable to impacts of climate change due to hazards such as changing water use patterns, shoreline erosion, sea level rise and storm surge [6].

The public health impacts of climate change in U.S. Gulf Coast states—Texas, Louisiana, Mississippi, Alabama, and Florida—may be especially severe and further exacerbated by a range of threats facing the coastline areas, including severe erosion, subsidence, and—given the amount of energy production infrastructure—the ever-present potential for large-scale industrial accidents. The Gulf Coast population is expected to reach over 74 million by 2030 [7] with a growing number of people living along the coastlines. Populations in the region that are already vulnerable because of economic or other disparities may face additional risks to health and well-being as a consequence of a changing climate, creating new levels of concern for political and public health leaders.

Although a growing body of research has focused on manifestations of climate change along the Gulf Coast and other parts of the Caribbean Basin (e.g., sea level rise, coastal erosion, saltwater intrusion, and extreme heat events), the potential impacts of climate variability and change on human health in the region have not been systematically investigated. Considering evidence from academic articles and reports published by governmental and non-governmental organizations, this paper provides an overview of present and projected conditions in the region followed by a discussion of adaptation strategies and recommendations. We start with an overview of projected climate change impacts in the Gulf Coast and the unique vulnerabilities of the region. Next, we discuss potential health impacts of climate variability and change in the region based on emerging research findings with a focus on the most relevant climate-sensitive hazards. We conclude with a discussion of adaptation strategies and recommendations for meeting the public health challenges the Gulf Coast may face in the coming decades.

## 2. Physical Climate and Projected Climate Change Impacts

**Figure 1:**
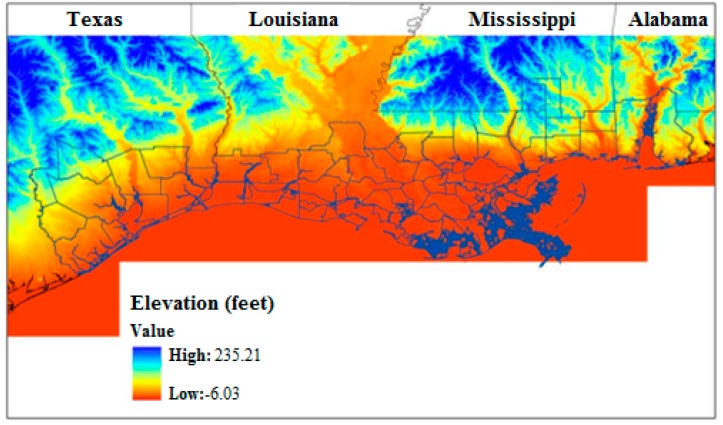
Relative elevation of Gulf Coast counties. Areas below 30 m elevation displayed in orange. Figure Source: Potter *et al.* [8].

Climate change impacts in the U.S. Gulf coast region are largely shaped by its coastal geography. As illustrated in Figure 1, a great portion of the region lies below 30 m elevation that makes it highly vulnerable to seasonal flooding, hurricanes, and tropical storms [8].

Subsidence, or the downward movement of the Earth’s surface [9,10], is a serious issue in the region that further exacerbates these vulnerabilities. A recent study of coastal Louisiana projected that spatially heterogeneous subsidence may consume nearly 50% of its coastal margin wetlands by 2100 [10]. Many parts of the Gulf coastline are also highly vulnerable to wetland loss [8].

The Gulf region is expected to experience increased mean temperatures and longer heat waves while freezing events are expected to decrease [11]. Regional average temperatures across the U.S. Southeast region (which includes Arkansas, Tennessee, Kentucky, Virginia, Georgia, North and South Carolina as well as the Gulf Coast) are projected to increase between 4 °F to 8 °F (2.2 °C to 4.4 °C) throughout the century [12]. Hurricanes and sea level rise, occurring independently or in combination with hurricane-induced storm surge, are major threats to the Gulf Coast region [11]. Some portions of the Gulf Coast—particularly coastal Louisiana and South Florida—are especially vulnerable to sea level rise due to their low elevation. Recent studies suggest that the Gulf Coast and Caribbean may experience fewer but more intense hurricanes [11]. The entire coastal region, and Charleston, Miami, New Orleans, Tampa and Virginia Beach, in particular, is vulnerable to more intense hurricane winds, rainfall, and storm surges [13]. According to another report, 70% of the Gulf of Mexico shoreline is susceptible to extreme erosion even during the weakest hurricanes [14]. Although sea level rise is likely inevitable, its speed is difficult to predict as it is influenced by a number of complex factors such as polar ice sheet dynamics [11].

## 3. U.S. Gulf Coast Vulnerability to Climate-Related Hazards

The Gulf Coast may suffer particularly severe consequences under a changing climate as a result of the concentration of people, including many socially disadvantaged populations, the regional infrastructure, and the unique vulnerabilities resulting from its coastal geography. Individual characteristics such as older age and race, and social vulnerability factors such as poverty, affect susceptibility to some climate-sensitive hazards like heat waves [15,16] and the ability to prepare for, respond to, or recover from others, such as hurricanes and other natural disasters [17].

The U.S. Gulf Coast states are projected to reach a combined population of 74.8 million by 2030 compared with 48.6 million as of 2000 [7], with a substantial proportion of the population living along the coastlines. Although only about 8% of the U.S. counties are located along saltwater coasts, they are home to 29% of the country’s population [18]. As of 2008, the 56 Gulf Coast coastline counties had a total population of over 8 million and experienced a 150% increase in population since 1960; a greater percentage increase compared to the two other U.S. coastal regions [18]. The Gulf Coast region is home to more people aged 65 years or older, as well as more blacks, compared to the U.S. average [19]. The U.S. Gulf coast is also home to large socially disadvantaged populations. For instance, 142 out of the 384 “persistent poverty counties” nationwide, defined as counties where 20% or more live in poverty, are located in the Gulf Coast states [20]. Black race, lower socioeconomic status, and being elderly are established risk factors for heat- and hurricane-related mortality [16,21].

Various manifestations of climate change can impact the Gulf Coast infrastructure, creating serious challenges to the health, well-being, and resilience of the affected communities. For instance, over the next 50–100 years, a sea-level rise of 4 feet (1.2 m)—1 to 4 feet/0.3 to 1.2 m of sea level rise that is projected globally by 2100 [1] could permanently inundate 2400 miles (3862 km) (27%) of the major roads between Mobile and the Houston/Galveston area—including roads designated as major evacuation routes—and 246 miles (396 km) of freight rail lines [22,23]. Coupled with hurricanes that would drop more rainfall and generate stronger winds and higher storm surges, such a rise in sea level would reduce the life expectancy of and increase maintenance and repair expenditures for roads, rail lines, pipelines, bridges, airports, and other transportation and communications systems on which the Gulf Coast economy depends [22]. Barrier island erosion and submersion render low-lying areas even more susceptible to the impacts of sea level rise.

Long-term land use patterns coupled with powerful hurricanes have contributed substantially to eroding barrier islands and wetlands that are coastal communities’ first line of defense against winds, waves, and storm surge. Coastal Louisiana has lost over 2400 square miles (6216 km^2^) of wetlands at an annual rate of 15 to 40 square miles (39 to 104 km^2^) since the 1930s [24], with more than 95% of the Louisiana shoreline affected by erosion [25]. About 85% of the surface of Chandeleur Islands, that serve as New Orleans’ first line of defense, was lost during Hurricane Katrina [8]. Studies increasingly acknowledge that Gulf Coast wetlands can play a role in protecting coastal communities from hurricane damage [26]. Nonetheless, if trends in loss rates continue, even in the presence of ongoing restoration efforts, Louisiana will lose more than 630,000 additional acres (254,952 hectares) of coastal wetland and islands by 2050 [27]. With barrier islands erosion and submersion and coastal wetlands loss, affected communities will have to strengthen their structural defenses, relocate, or face increasing risks [25].

The vulnerability of the Gulf coast to climate-related hazards is reflected in the costs the region is projected to face in the coming decades. Between 1980 and 2012, the Southeast was the U.S. region affected by the greatest number of billion-dollar disasters and Texas was affected by more billion-dollar disasters than any other state [12]. The physical impacts of climate change in Louisiana are likely to be especially severe and the state is expected to face billions of dollars in increased disaster costs as early as 2030. The Gulf Coast currently experiences average annual losses of $14 billion from hurricane winds, land subsidence, and sea level rise; losses by 2030 could reach $18 billion (no climate change scenario) to $23 billion (extreme climate change scenario) [12,28].

## 4. Public Health Impacts

Climate change may amplify existing public health impacts, as well as introduce new health hazards to the Gulf Coast. In most instances climate change amplifies the risk for existing health impacts such as heat-related morbidity and mortality, malnutrition resulting from droughts, and injury and deaths following exposure to floods [29]. The increased frequency of extreme events and gradual changes in temperature and sea level can have impacts on human health. Social characteristics such as poverty or living alone increase the risk of morbidity and mortality associated with some climate-sensitive hazards such as high temperatures [15,16]. In the case of natural disasters, population characteristics such as age and income have a substantial influence on the capacity for population preparedness, response, and recovery [17]. Climate change will disproportionately affect already vulnerable communities including children, the elderly, racial minorities, those of lower socioeconomic status, and those with pre-existing health conditions [1].

### 4.1. Health Impacts of Hurricanes, Floods and Storms

Over the past decade, the Gulf Coast population suffered greatly from hurricanes, floods, and storms [30]. Hurricane Katrina, the costliest and one of the deadliest hurricanes in U.S. history [21,31], exposed the vulnerability of the public health system to large-scale disasters. With tens of thousands of residents of Mississippi, Louisiana, and Alabama left without access to basic health care, public health leaders emphasized the vulnerability and lack of resources to address the needs of the poor in the affected areas during Katrina, as well as to future disasters [32]. Projected sea level rise and more intense hurricanes may contribute to challenges to public health and emergency response and recovery.

Loss of life is the most severe and easily attributable impact of extreme weather events. The U.S. Gulf coast has been hard hit by some of the deadliest hurricanes in history, with thousands of hurricane-related deaths documented in the 20th century [30]. Hurricane Katrina caused approximately 1500 deaths with the greatest number of victims documented in Louisiana followed by Mississippi [31]. In addition to the deaths directly attributed to Katrina, additional Katrina-related deaths have likely occurred in the years following the event as a result of the increased incidence of physical and psychological problems including post-traumatic stress disorder, and widespread sense of despair and isolation among the victims [33,34]. In Louisiana, drowning, injuries or traumas, and heart conditions were the leading causes of Katrina-related mortality, with individuals aged 75 years and over and blacks more likely to be affected [21]. With hurricane intensity projected to increase with the changing climate, the large numbers of retirement age and black individuals in the Gulf Coast may suffer disproportionately from hurricane-related mortality.

Outbreaks inevitably follow large-scale disasters when public health systems are affected. For instance, the number of reported cases of West Nile disease increased substantially in Louisiana and Mississippi after Hurricane Katrina [35], and an outbreak of norovirus was documented among evacuees in Texas [36]. Although the immediate health impacts of natural disasters have the most devastating impacts on communities and receive the most attention, they are often only a precursor to long term physical and mental health impacts among some affected individuals for years and even decades [37,38,39]. Numerous studies among Hurricane Katrina survivors reported persisting physical and mental health issues [40,41,42]. For example, the National Comorbidity Survey-Replication allowed comparison of mental health outcomes post-Katrina in the same area where the survey was administered between 2001 and 2003. Respondents to the post-Katrina survey reported a significantly higher prevalence of serious and mild-moderate mental illness [40]. In addition, several decades of studies conducted in the aftermath of disasters provide evidence of a substantial burden of post-traumatic stress disorder among persons who experience a disaster, with the extent of exposure to a disaster being the most important risk factor for the development of disaster-related PTSD in many instances [39]. Another study discussed the strong associations of hurricane-related stressors with high prevalence of anxiety-mood disorders that was independent of socio-demographics [42]. Psychological morbidity, in particular following hurricanes, increases in children and adults, then declines over time, depending on preexisting mental health status [43].

### 4.2. Temperature-Related Health Impacts

Heat-related mortality and morbidity are the most well understood and easily measureable impacts of changing weather patterns on human health. The very young and the elderly, individuals of low socioeconomic status, and those with preexisting medical conditions, particularly cardiovascular and respiratory, are at increased risk [15,16]. Most studies in the U.S. have focused on quantifying heat-related mortality as a result of elevated ambient temperatures or heat waves [44]. Elevated ambient temperatures increase total, cardiovascular, and respiratory morbidity [45], emergency department visits, and ambulance calls for heat-related illnesses during or after a heat event [46,47].

The risk of heat-related mortality varies across the United States [48,49,50], with people living in the South better acclimatized to high temperatures compared with those living in the Northeast or Midwest, and thus generally less susceptible to the impacts of heat [48]. Although mortality impacts of heat have been declining over time due to adaptation, even communities with lower mortality risks attributable to heat will likely continue to experience adverse health effects due to rising temperatures [44,51]. With rising average temperatures and longer heat waves projected in the Gulf Coast over the coming decades, heat-related fatalities may occur particularly among susceptible populations such as the elderly and those living in poverty. At the moment, 14% of the Gulf Coast region population is over the age of 65 compared with 13% nationwide, and 17% of the population lives under the poverty level compared with 13% nationwide.

A recent study projected the impacts of climate change on extreme heat event attributable deaths in the United States, including projections for numerous Gulf Coast cities: Jacksonville, Tampa and Miami, Florida, New Orleans, Louisiana, San Antonio, Dallas and Houston, Texas and Birmingham, Alabama [52]. By the end of the twenty-first century, all Gulf Coast cities included in the study are projected to experience an increase in the average number of extreme heat event days compared with the 1975–1995 baseline. Because extreme heat events are defined relative to the local climate, cities with minimal summer weather variability such as Miami and Houston do not have a large number of extremely hot days and do not experience substantial heat-related mortality despite having relatively hot climates. Nonetheless, the study projected that heat-related deaths will increase over time in all cities except Miami under the A1 emission scenario (medium-high emissions) developed by the Intergovernmental Panel on Climate Change (IPCC) Special Report on Emission Scenarios (SRES) [52].

### 4.3. Vector-Borne Diseases

The incidence of some vector-and water-borne diseases may increase under a changing climate. In particular, climate change may lead to the expansion of the tropical wet-dry climate zone where conditions for vector-borne disease transmission are most favorable [53]. Among the studies highlighting climate change’s potential to change the geographic range of known vectors, some discussed the reappearance in the United States—particularly in the southeast—of dengue fever [54,55]. In a recent review of dengue historic outbreaks and reemergence in the United States, Beaumier and colleagues concluded that periodic outbreaks of dengue fever should be expected in high risk areas, noting that between 2010 and 2013, 87 dengue cases were reported in Florida and three in Texas [56]. Most malaria cases in the United States are currently imported by travelers to areas where malaria is endemic [57]. For instance, there were 94 cases contracted in Haiti that were reported in Florida in 2010 and 25 cases in 2011 [58]. Outbreaks of locally acquired cases still occur in the region [57,59]. Although future trends are difficult to project, warmer temperatures, humidity, and access to water may alter vector-borne transmission cycles and facilitate the re-introduction of vector-borne diseases such as malaria, yellow fever, and dengue fever, that have been mostly eradicated in the Gulf region.

### 4.4. Migration

Climate change and human migration have been linked for thousands of years with social, demographic, or economic factors that are major driving forces behind migration [60]. Environmental factors also play an important role in influencing migration decisions among vulnerable populations [61].

Migration may have severe and lasting mental health effects on affected populations due to their place attachment. Place attachment is a bond between people and their natural environment, based on cognition and affect [62]. The subsequent loss of this bond can have a negative impact on mental health. In this regard, the issues surrounding Hurricane Katrina offer a prominent warning of what the future may hold. About 1.5 million Gulf Coast residents were evacuated, with thousands still displaced [63]. By 2010, the population of the New Orleans region declined by 25.4% and migrants dispersed throughout the United States with as many as 250,000 in Texas alone [64]. Thus, the psychological issues associated with migration must receive consideration in assessing climate change impacts.

Additionally, few studies address the possibility that the Gulf Coast might receive new inhabitants from other impacted nations in the Caribbean Basin. As the Foresight report on migration and global environmental change [64] noted, migration is likely to continue despite environmental change trends, and individuals are as likely to migrate to places of environmental vulnerability as from such places. Large-scale migration, particularly on an international scale may be exacerbated under climate change and will likely pose security challenges. Migration and permanent relocation of large numbers of people will put additional pressures on already stressed vital health and social services, including affordable housing, on the destination communities, where, for instance, access to health care might be reduced for all sectors of the population. This would apply to the newly migrated, as well as long-standing residents [60]. Projected changes in the incidence of extreme events may amplify the health risks of economically disadvantaged migrants from and to urban centers (IPCC 2014). For instance, higher income New Orleans residents were able to migrate proactively and better adapt to the challenges following Hurricane Katrina while the economically disadvantaged populations were likely to be involuntarily displaced and lacked the resources necessary for adaptation [63,64,65].

## 5. Recommended Public Health Adaptation Measures

The goal of adaptation is to increase the capacity of individuals and communities to prepare for and manage the health risks of climate change. These policies and measures can contribute to community and regional resiliency, and are more effective when instituted as part of broad investment strategies that strengthen a community’s ability to manage change.

The basic tenets and tools of the public health discipline are up to the challenges of climate change [3,29,66,67,68,69]. Public health adaptation is analogous to public health preparedness as it aims to reduce the health burden of climatic changes on society [67]. A wide range of short- and long-term adaptation strategies can increase resilience to climate-sensitive health outcomes. Adaptation can be viewed as autonomous or planned depending on whether it results from reactive or anticipatory actions [4]. For instance, migration as a result of a major hurricane is an example of an autonomous adaptation. The implementation of a hurricane warning system is, in contrast, an example of planned adaptation. Because autonomous adaptation is likely unable to adequately address the health challenges posed by a changing climate, the need for planned adaptation, aimed at increasing resilience, is widely recognized. The Third National Climate Assessment highlights the need for creating the “strongest climate-health preparedness programs possible” in the United States by investing in programs that are effective in addressing current public health threats expected to worsen with climate change [1].

Climate change is also increasingly acknowledged as a threat to national security and there is a growing political support for actions to build resilience and simultaneously minimize possible health-related impacts [70]. In 2013, President Obama issued a Climate Action Plan followed by an Executive Order to create a Task Force on Climate Preparedness and Resilience of state, local, and tribal officials [71], followed by a $1 billion initiative to help communities undertake climate change resilience improvements. The Task Force’s national areas of focus include food, coastal drought, wildland fire and climate resilience as well as future disaster mitigation [72]. In the U.S. Gulf Coast, climate change resilience improvements are of critical importance for protecting public health in light of projected sea level rise and the potential for weather-related catastrophes.

Based on our overview of the potential public health impacts the Gulf Coast states may face in the coming decades, we recommend a number of adaptation measures that could be implemented, preferably in the context of an overall climate adaptation plan to improve the resilience of individuals, health systems and infrastructure (Table 1) but that could be implemented independently where necessary. Integrating adaptation at the city, state and regional level can greatly enhance hazard-specific adaptation measures.

Measures to address the underlying vulnerabilities of children, the elderly, racial minorities, those with lower socioeconomic status, and those with pre-existing medical conditions should be of primary importance because such individuals will likely suffer disproportionately from the impacts of changing weather patterns due to climate change. Developing risk communication and educational programs for vulnerable populations and focusing on such populations during extreme weather events could substantially reduce climate-related mortality and morbidity. The state of Florida has been particularly effective in targeting vulnerable ageing populations [73]. For instance, the Florida Department of Elder Affairs works closely with the state’s emergency operations center during disasters to determine if area agencies on aging need special assistance. The state also uses census data and geographic maps to efficiently allocate resources in areas with greater concentration of older adults, thus allowing volunteers and social workers to focus on better addressing the needs of older adults [73].

**Table 1:** Proposed adaptation measures.

Health Impact	Adaptation Measures
Health impacts of extreme events	Improve health system preparedness to extreme weather events by implementing new and improving existing warning systems Ensure supply chains Strengthen infrastructure resilience to sea level rise Modify building codes to incorporate information about potential risks under climate change Continuously update and improve emergency risk communication strategies with special focus on outreach to vulnerable populations Education and capacity building
Temperature-related health impacts	Facilitate access to community education programs about heat risks and promote better understanding of heat warnings and awareness about resources available during heat waves Operate cooling centers during heat events Develop air conditioning programs for low income households and individuals at risk for heat-related illness Improve regulation of indoor temperature by implementing green/white roofs and other measures Education and capacity building
Vector-borne diseases	Implement surveillance systems for climate sensitive diseases Systematically collect baseline information on disease prevalence Enhance health system capacity for rapid disease-specific emergency response
Migration	Introduce long term programs to prevent future long distance migration by providing resettlement opportunities from risk areas (e.g. coastline regions highly vulnerable to sea level rise) Assure timely relocation of displaced individuals and maintenance of social structure to the greatest extent possible when forced migration occurs

Investments in strengthening public health systems are of critical importance, especially concerning their resilience to climate-sensitive hazards and capacity for timely emergency response. In this regard, focusing on new and existing programs that address public health threats that may worsen with climate change such as heat wave early warning systems is likely to be particularly effective. Successful initiatives to improve the capacity of Gulf Coast communities to prepare for and respond to climate-sensitive hazards were developed in recent years. For example, the Sea-Grant Mississippi-Alabama consortium funded a Climate Community of Practice that assembled a group of extension, outreach, and education professionals in the Gulf. This group works together with community members and officials to provide education and enhance the capacity of coastal communities to adapt to sea-level rise and other climate-related issues [74]. Adaptation strategies are ultimately incorporated into the Gulf Coast communities’ resilience plans.

Investments in improving infrastructure resilience are likely to pay off over time given the losses from climate-sensitive weather events the Gulf Coast is already suffering; these investments also can reduce mortality and morbidity, and the public health consequences from migration. Efforts are underway to improve infrastructure resilience in the region by taking into consideration historical data and projections. For example, following Hurricanes Katrina and Rita in 2005, Louisiana adopted a Comprehensive Master Plan for a Sustainable Coast with the purpose of coordinating local, state, and federal efforts to achieve long term coastal protection and restoration [75]. In particular, the Plan aims to reduce storm surge flood risks to coastal communities and implement other measures to improve the coast’s sustainability over the next 50 years, with a focus on communities, expanding partnerships, and improving models using the best available science [76]. Analytical models, such as the analytical Louisiana Risk Assessment (CLARA) model developed by RAND that estimates flood depths and damage occurring as a result of major storms, allow the systematic evaluation of projects to be included in the Master Plan based on their promise to minimize flood damage in Louisiana’s coastal region [77]. The model derives projections for a “future without action” and a “future with the final Master Plan in place” in 2012, 2036, and 2061, estimating the annual likelihood of storm surge flooding and impacts on different infrastructure options under various sea-level rise and coastal subsidence scenarios. For instance, by 2061, expected annual flood damage could be reduced from over US$20 billion to around 4 billion if actions recommended in the Master plan were implemented.

## 6. Conclusions

With climate change already underway, the U.S. Gulf Coast may increasingly experience extreme events such as hurricanes, floods, and storms that can in turn exacerbate the associated health impacts in the region, particularly among the most vulnerable. The Gulf Coast’s vulnerability is further exacerbated due to its large population, substantial amount of at-risk infrastructure and unique coastal geography. In addition, the region is home to large populations of socially disadvantaged individuals that are likely to suffer disproportionately from the impacts of changing weather patterns and sea level rise. As demonstrated in the aftermath of large-scale disasters such as Hurricane Katrina, socioeconomic factors can amplify their devastating impacts. Echoing an observation from the disaster research literature, the world’s poor and powerless are likely to suffer not just disproportionately from climate change, but fatally [78]. Public health adaptation can substantially enhance the capacity of the region to address climate-sensitive health hazards in the future. A comprehensive adaptation strategy for the Gulf encompassing measures to improve individual, public health system, and infrastructure resilience is essential. Such measures will help reduce the threats to population health posed by rapid climate change in one of the Unites States’ most vulnerable regions.

## References

[1] Luber G., Knowlton K., Balbus J., Frumkin H., Hayden M., Hess J., McGeehin M., Sheats N., Backer L., Beard C.B., Melillo J.M., Richmond T.T.C., Yohe G.W. (2014). Human Health. Climate Change Impacts in the United States: The Third National Climate Assessment.

[2] Costello A., Abbas M., Allen A., Ball S., Bell S., Bellamy R., Friel S., Groce N., Johnson A., Kett M. (2009). Managing the health effects of climate change. Lancet.

[3] Luber G., Prudent N. (2009). Climate change and human health. Trans. Am. Clin. Climatol. Assoc..

[4] Field C.B., Barros V.R., Dokken D.J., Mach K.J., Mastrandrea M.D., Bilir T.E., Chatterjee M., Ebi K.L., Estrada Y.O., Genova R.C., IPCC (2014). Climate Change 2014: Impacts, Adaptation, and Vulnerability. Part A: Global and Sectoral Aspects. Contribution of Working Group II to the Fifth Assessment Report of the Intergovernmental Panel on Climate Change.

[5] Brown O. (2008). Migration and Climate Change.

[6] Moser S.C., Davidson M.A., Kirshen P., Mulvaney P., Murley J.F., Neumann J.E., Petes L., Reed D., Melillo J.M., Richmond T.T.C., Yohe G.W. (2014). Coastal Zone Development and Ecosystems. Climate Change Impacts in the United States: Coastal Zone Development and Ecosystems. Climate Change Impacts in the United States: The Third National Assessment.

[7] U.S. Census Bureau, Population Division, Population Projections Branch Interim Projections: Ranking of Census 2000 and Projected 2030 State Population and Change: 2000 to 2030. http://www.census.gov/population/www/projections/popproj.html.

[8] Potter J.R., Burkett V.R., Savonis M.J., Potter J.R., Burkett V.R., Savonis M.J., Dokken D.J. (2008). Impacts of Climate Change and Variability on Transportation Systems and Infrastructure: Gulf Coast Study, Phase I.

[9] Dokka R.K. (2006). Modern-day tectonic subsidence in coastal Louisiana. Geology.

[10] Kent J., Dokka R. (2013). Potential impacts of long-term subsidence on the wetlands and evacuation routes in coastal Louisiana. GeoJournal.

[11] Biasutti M., Sobel A.H., Camargo S.J., Creyts T.T. (2012). Projected changes in the physical climate of the Gulf Coast and Caribbean. Clim. Chang..

[12] Carter L.M., Jones J.W., Berry L., Burkett V., Murley J.F., Obeysekera J., Schramm P.J., Wear D., Melillo J.M., Richmond T.T.C., Yohe G.W. (2014). Southeast and the Caribbean. Climate Change Impacts in the United States: The Third National Climate Assessment.

[13] Strauss B.H., Ziemlinski R., Weiss J.L., Overpeck J.T. (2012). Tidally adjusted estimates of topographic vulnerability to sea level rise and flooding for the contiguous United States. Environ. Res. Lett..

[14] Stockdon H.F., Doran K.J., Thompson D.M., Sopkin K.L., Plant N.G., Sallenger A.H. (2012). National Assessment of Hurricane-Induced Coastal Erosion Hazards—Gulf of Mexico: U.S. Geological Survey Open-File Report 2012–1084.

[15] Basu R., Samet J.M. (2002). Relation between elevated ambient temperature and mortality: A review of the epidemiologic evidence. Epidemiolo. Rev..

[16] Basu R. (2009). High ambient temperature and mortality: A review of epidemiologic studies from 2001 to 2008. Environ. Health.

[17] Oxfam America (2009). Exposed: Social Vulnerability and Climate Change in the US Southeast.

[18] Wilson S.G., Fischetti T.R. (2010). Coastline Population Trends in the United States: 1960 to 2008.

[19] NOAA (2011). The Gulf of Mexico at a Glance: A Second Glance.

[20] United States Department of the Treasury (2012). Community Development Financial Institutions Fund: Persistent Poverty Data—By County. http://www.cdfifund.gov/what_we_do/persistentpoverty.asp.

[21] Brunkard J., Namulanda G., Ratard R. (2008). Hurricane Katrina deaths, Louisiana, 2005. Disaster Med. Public Health Prep..

[22] Karl T.R., Melillo J.M., Peterson T.C. (2009). Global Climate Change Impacts in the United States.

[23] Savonis M.J., Burkett V.R., Potter J.R. Impacts of Climate Change and Variability on Transportation Systems and Infrastructure: Gulf Coast Study, Phase I.

[24] CRPA (2010). Fiscal Year 2011 Annual Plan: Integrated Ecosystem Restoration and Hurricane Protection in Coastal Louisiana.

[25] USACE (2009). Louisiana Coastal Protection and Restoration.

[26] Barbier E.B., Georgiou I.Y., Enchelmeyer B., Reed D.J. (2013). The value of wetlands in protecting southeast Louisiana from hurricane storm surges. PLoS ONE.

[27] (1998). Louisiana Coastal Wetlands Conservation and Restoration Task Force and the Wetlands Conservation and Restoration Authority. Coast 2050: Toward a Sustainable Coastal Louisiana.

[28] (2010). America’s Wetland Foundation; America’s Energy Coast; Entergy (AWF/AEC/Entergy). Building a Resilient Energy Gulf Coast: Executive Report.

[29] Frumkin H., Hess J., Luber G., Malilay J., McGeehin M. (2008). Climate change: The public health response. Am. J. Public Health.

[30] National Weather Service. http://www.weather.gov/.

[31] Beven J.L., Avila L.A., Blake E.S., Brown D.P., Franklin J.L., Knabb R.D., Pasch R.J., Rhome J.R., Stewart S.R. (2008). Atlantic hurricane season of 2005. Mon. Wea. Rev..

[32] The New York Times (2005). Health Care for Katrina Victims. http://www.nytimes.com/2005/10/04/opinion/04tue2.html?_r=0.

[33] Faust K.L., Kauzlarich D. (2008). Hurricane Katrina victimization as a state crime of omission. Crit. Crim..

[34] Mutter J. (2008). Hurricane Katrina Deceased Victim List. http://www.katrinalist.columbia.edu.

[35] Caillouët K.A., Michaels S.R., Xiong X., Foppa I., Wesson D.M. (2008). Increase in West Nile neuroinvasive disease after Hurricane Katrina. Emerg. Infect. Dis..

[36] CDC (2005). Infectious disease and dermatologic conditions in evacuees and rescue workers after Hurricane Katrina—Multiple states, August–September, 2005. Morb. Mortal. Wkly. Rep..

[37] Rubonis A.V., Bickman L. (1991). Psychological impairments in the wake of disaster: The disaster-psychopathology relationship. Psychol. Bull..

[38] Solomon S.D., Green B.L. (1992). Mental health effects of natural and human-made disasters. PTSD Res. Q..

[39] Galea S., Nandi A., Vlahov D. (2005). The epidemiology of post-traumatic stress disorder after disasters. Epidemiol. Rev..

[40] Kessler R.C., Galea S., Jones R.T., Parker H.A. (2006). Mental illness and suicidality after Hurricane Katrina. Bull. World Health Organ..

[41] Wang P.S., Gruber M.J., Powers R.E., Schoenbaum M., Speier A.H., Wells K.B., Kessler R.C. (2007). Mental health service use among Hurricane Katrina survivors in the eight months after the disaster. Psychiatr. Serv..

[42] Galea S., Brewin C.R., Gruber M., Jones R.T., King D.W., King L.A., McNally R.J., Ursano R.J., Petukhova M., Kessler R.C. (2007). Exposure to hurricane-related stressors and mental illness after Hurricane Katrina. Arch. Gen. Psychiatry.

[43] Bourque L.B., Siegel J.M., Kano M., Wood M.M. (2006). Weathering the storm: The impact of hurricanes on physical and mental health. Ann. Am. Acad. Polit. Soc. Sci..

[44] Petkova E.P., Morita H., Kinney P.L. (2014). Health impacts of heat in a changing climate: How can emerging science inform urban adaptation planning?. Curr. Epidemiol. Rep..

[45] Ye X.F., Wolff R., Yu W.W., Vaneckova P., Pan X.C., Tong S.L. (2012). Ambient Temperature and Morbidity: A Review of Epidemiological Evidence. Environ. Health Perspect..

[46] Lippmann S.J., Fuhrmann C.M., Waller A.E., Richardson D.B. (2013). Ambient temperature and emergency department visits for heat-related illness in North Carolina, 2007–2008. Environ. Res..

[47] Hartz D.A., Golden J.S., Sister C., Chuang W.C., Brazel A.J. (2012). Climate and heat-related emergencies in Chicago, Illinois (2003–2006). Int. J. Biometeorol..

[48] Anderson B.G., Bell M.L. (2009). Weather-Related Mortality How Heat, Cold, and Heat Waves Affect Mortality in the United States. Epidemiology.

[49] Anderson G.B., Bell M.L. (2011). Heat Waves in the United States: Mortality Risk during Heat Waves and Effect Modification by Heat Wave Characteristics in 43 U.S. Communities. Environ. Health Perspect..

[50] Medina-Ramon M., Schwartz J. (2007). Temperature, temperature extremes, and mortality: A study of acclimatisation and effect modification in 50 US cities. Occup. Environ. Med..

[51] Bobb J.F., Peng R.D., Bell M.L., Dominici F. (2014). Heat-related mortality and adaptation to heat in the United States. Environ. Health Perspect..

[52] Greene S., Kalkstein L.S., Mills D.M., Samenow J. (2011). An examination of climate change on extreme heat events and climate-mortality relationships in large U.S. cities. Weather. Clim. Soc..

[53] Ingram K., Dow K., Carter L., Anderson J. (2013). Climate of the Southeast United States: Variability, Change, Impacts, and Vulnerability.

[54] Barclay E. (2008). Is climate change affecting dengue in the Americas?. Lancet.

[55] Morens D.M., Fauci A.S. (2008). Dengue and hemorrhagic fever: A potential threat to public health in the United States. J. Am. Med. Assoc..

[56] Beaumier C., Garcia M.N., Murray K.O. (2014). The History of Dengue in the United States and its Recent Emergence. Curr. Trop. Med. Rep..

[57] CDC (2003). Local transmission of Plasmodium vivax malaria—Palm Beach County, Florida, 2003. Morb. Mortal. Wkly. Rep..

[58] Cullen K.A., Arguin P.M. (2013). Malaria Surveillance—United States, 2011. MMWR Surveill. Summ..

[59] Filler S.J., MacArthur J.R., Parise M., Wirtz R., Eliades M.J., Dasilva A., Steketee R. (2006). Locally Acquired Mosquito-Transmitted Malaria: A Guide for Investigations in the United States.

[60] Smith P.J. (2007). Climate change, mass migration and the military response. Orbis.

[61] Hunter L. (2005). Migration and Environmental Hazards. Popul. Environ..

[62] Stedman R.C. (2002). Toward a social psychology of place predicting behavior from place-based cognitions, attitude, and identity. Environ. Behav..

[63] Weber L., Peek L. (2012). Displaced: Life in the Katrina diaspora.

[64] (2011). Foresight: Migration and Global Environmental Change.

[65] Mutter J. (2010). Disasters widen the rich-poor gap. Nature.

[66] Ebi K.L., Kovats R.S., Menne B. (2006). An approach for assessing human health vulnerability and public health interventions to adapt to climate change. Environ. Health Perspect..

[67] Ebi K.L., Semenza J.C. (2008). Community-based adaptation to the health impacts of climate change. Am. J. Prev. Med..

[68] Ebi K. (2011). Climate change and health risks: Assessing and responding to them through “adaptive management”. Health Aff..

[69] Woodward A., Smith K.R., Campbell-Lendrum D., Chadee D.D., Honda Y., Liu Q.Y., Olwoch J., Revich B., Sauerborn R., Chafe Z. (2014). Climate change and health: On the latest IPCC report. Lancet.

[70] Board C.M.A. (2014). National Security and the Accelerating Risks of Climate Change. http://www.cna.org/sites/default/files/MAB_2014.pdf.

[71] White House Executive Order. https://www.whitehouse.gov/the-press-office/2013/11/01/executive-order-preparing-united-states-impacts-climate-change.

[72] Environmental and Energy Institute Issue Brief (2015). White House Task Force on Climate Preparedness and Resilience Recommended Actions. http://www.ourenergypolicy.org/wp-content/uploads/2015/04/IssueBrief_White_House_Climate_Task_Force_Recommendations_040115.pdf.

[73] Centers for Disease Control and Prevention (CDC) CDC’s Disaster Planning Goal: Protect Vulnerable Older Adults. http://www.cdc.gov/Aging/pdf/disaster_planning_goal.pdf.

[74] Mississippi-Alabama Sea Grant Consortium Climate Outreach Community of Practice. http://masgc.org/climate-outreach-community-of-practice/summary.

[75] Coastal Protection and Restoration Authority Master Plan Overview. http://coastal.la.gov/a-common-vision/master-plan/.

[76] Coastal Protection and Restoration Authority 2017 Coastal Master Plan Update. http://coastal.la.gov/a-common-vision/2017-master-plan-update/.

[77] RAND Coastal Louisiana Risk Assessment Model. http://www.rand.org/pubs/technical_reports/TR1259.html.

[78] Canadian Medical Association Journal (CMAJ) (2005). Katrina, climate change and the poor. Can. Med. Assoc. J..

